# P-1161. Identifying Resilient Performance in Outpatient Hemodialysis During COVID-19: A Longitudinal Cluster Analysis of BSI and IVAS Rates

**DOI:** 10.1093/ofid/ofaf695.1354

**Published:** 2026-01-11

**Authors:** Lacey Critchley, Rahsaan Overton, Lu Meng, Qunna Li, Jonathan R Edwards, Jeneita Bell, Shannon Novosad, Sarah H Yi, Matthew J Stuckey

**Affiliations:** CDC (contractor), Atlanta, Georgia; Centers for Disease Control and Prevention, Atlanta, GA; CDC, Atlanta, Georgia; CDC, Atlanta, Georgia; Centers for Disease Control and Prevention, Atlanta, GA; Centers for Disease Control and Prevention, Atlanta, GA; Centers for Disease Control and Prevention, Atlanta, GA; Centers for Disease Control and Prevention, Atlanta, GA; Division of Healthcare Quality Promotion, Centers for Disease Control and Prevention, Atlanta, Georgia

## Abstract

**Background:**

The COVID-19 pandemic underscored the importance of resilient, safe care delivery in healthcare systems during times of stress. Bloodstream infections (BSIs) remain a major cause of death among hemodialysis (HD) patients, making safe care essential, especially during disruption. This study used a novel approach to characterize resilient performance patterns in US outpatient HD facilities during the COVID-19 pandemic.

**Figure:**
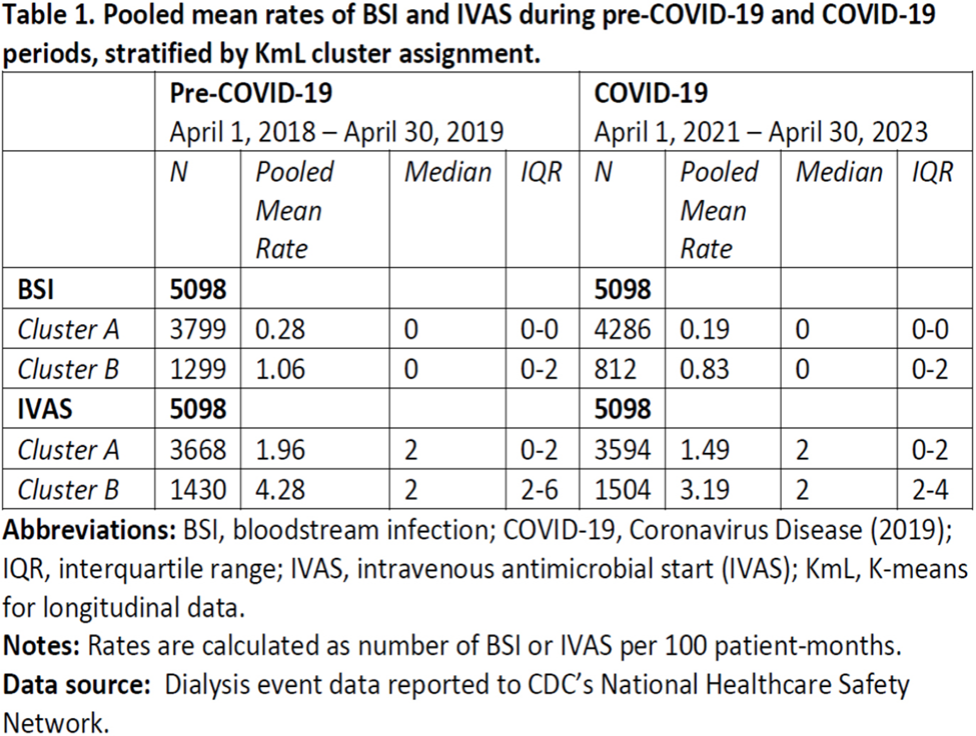

**Figure:**
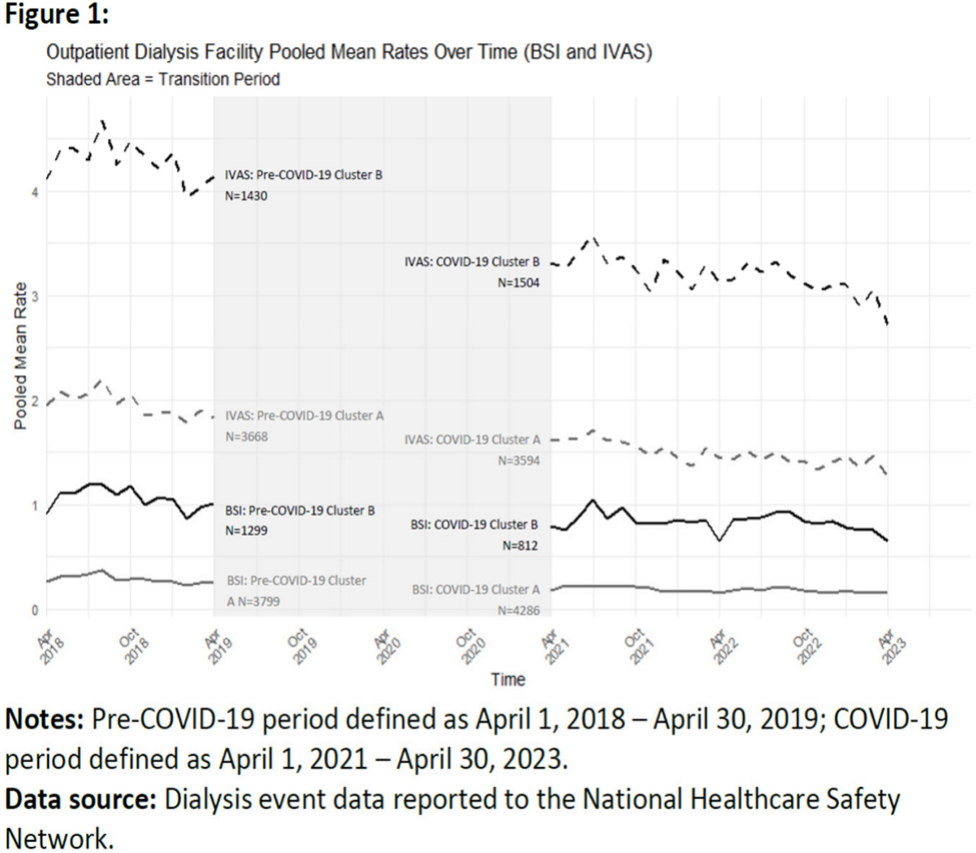

**Methods:**

Using a retrospective longitudinal design, we analyzed rates of two patient safety measures—BSI and intravenous antimicrobial starts (IVAS)—reported to the National Healthcare Safety Network (NHSN) before and during the COVID-19 pandemic. Inclusion criteria were outpatient HD facilities serving adults with complete BSI and IVAS reporting during both pre-COVID-19 (April 2018–April 2019) and COVID-19 (April 2021–April 2023) periods, plus complete annual survey and COVID-19 reporting. We applied K-means for longitudinal data (KmL), a machine-learning method, to classify facilities into clusters based on BSI and IVAS rates in each period. Resilient performance was assessed by concordance in cluster assignments between periods using percent agreement and McNemar’s test.

**Figure:**
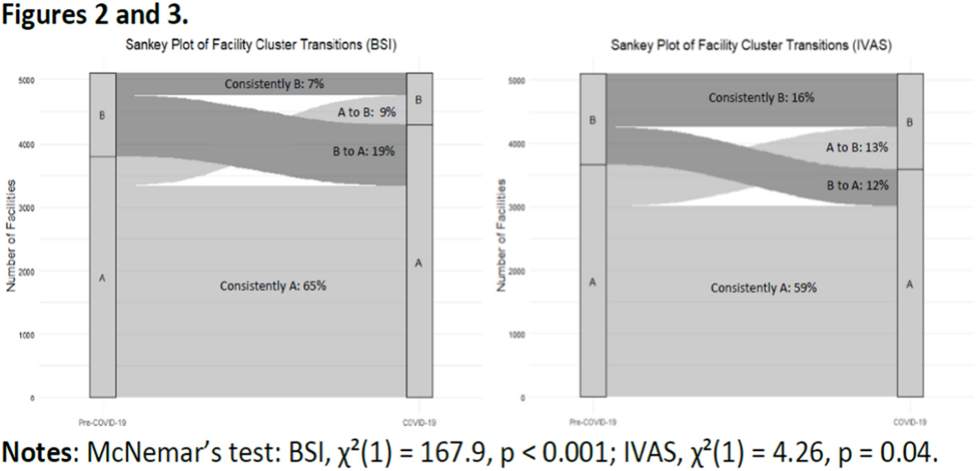

**Results:**

Of 7052 facilities reporting to NHSN, 5098 (72%) met inclusion criteria. For each outcome, KmL assigned facilities into one of two clusters (A or B) per period (Table 1, Figure 1). On average, facilities in cluster A had lower pooled mean rates and cluster B had higher rates. For BSI, 9% of facilities in cluster A pre-COVID-19 transitioned to cluster B during COVID-19, while 19% shifted from cluster B to cluster A (Figure 2). For IVAS, 13% transitioned to cluster B and 12% to cluster A (Figure 3). McNemar’s test revealed asymmetry in transitions.

**Conclusion:**

We identified different facility-level patterns in BSI and IVAS outcomes before and during COVID-19. These methods may help healthcare systems and public health identify resilient performance patterns during system stress. Identifying facilities where rates increased may help target additional support and resources. With a focus on facilities that shifted between clusters, future analyses will identify operational factors associated with the resilient delivery of safe care.

**Disclosures:**

All Authors: No reported disclosures

